# Pharmacy Students’ Perceptions of Remote versus Face-to-Face Learning Experience

**DOI:** 10.3390/pharmacy11030097

**Published:** 2023-06-08

**Authors:** Jenna M. Mills, Celeste N. VanAtta, Racheal S. Hendershot, Shantanu Rao

**Affiliations:** 1 Department of Pharmacy Practice, College of Pharmacy, University of Findlay, Findlay, OH 45840, USA; celeste.vanatta@findlay.edu; 2 College of Pharmacy, University of Findlay, Findlay, OH 45840, USA; hendershotr@findlay.edu; 3 Department of Pharmaceutical Sciences, College of Pharmacy, University of Findlay, Findlay, OH 45840, USA; rao@findlay.edu

**Keywords:** remote learning, student motivation, engagement, COVID-19

## Abstract

During the COVID-19 pandemic, there was a large shift from face-to-face (FTF) to remote learning. Evaluating students’ perceptions of remote learning provides educators with opportunity to inform their instructional methods. This study sought to evaluate pharmacy students’ self-perceived (1) confidence, (2) preparedness, (3) satisfaction, and (4) motivation following remote vs. FTF classes. An electronic survey was distributed to six pharmacy student cohorts enrolled in the University of Findlay College of Pharmacy during April 2021 to measure the objectives. The Kruskal–Wallis, Mann–Whitney U, and Spearman’s rank correlation tests were used to analyze the data (alpha = 0.05). A total of 151 students completed the survey. While the responses differed among the cohorts, first-professional year students reported lower motivation to study (*p* = 0.008), engage (*p* = 0.008), satisfaction with content presentation (*p* = 0.05), preparedness for exams (*p* < 0.001), and confidence to communicate (*p* = 0.008) and succeed in a career (*p* < 0.001) when studying remotely vs. taking FTF classes compared to fourth-professional year students. Positive correlations were observed between students who felt motivated to engage and study (ρ = 0.501, *p* < 0.001), motivated to study and exam preparedness (ρ = 0.511, *p* < 0.001), satisfied with course material presentation and professor accessibility (ρ = 0.688, *p* < 0.001), and exam preparedness (ρ = 0.521, *p* < 0.001), and felt prepared for exams and able to succeed in a pharmacy career (ρ = 0.573, *p* < 0.001). Taking the above results into consideration, pharmacy educators may designate more time and instructional support to first-professional year students in an effort to improve students’ perceptions of motivation, satisfaction, confidence, and preparedness.

## 1. Introduction

Traditionally, Doctor of Pharmacy (PharmD) programs have been offered in a face-to-face (FTF) manner, until the turn of the 21st century, when the first PharmD distance learning program was accredited in the United States [1]. Since the inception of this innovative program in 2001, there have been only a handful of other institutions that have followed suit. Fast forward to the early months of the COVID-19 pandemic, and many universities closed their campuses and switched to remote platforms to comply with social distancing practices. This sudden shift to distance learning resulted in PharmD programs transitioning their FTF lecture content to an online platform for remote learners [2]. With 14,000+ new PharmD graduates entering the workforce each year, it may be of interest for pharmacy educators to gauge how students perceived the impact of remote learning on their education and future pharmacy careers [3]. Moreover, as additional PharmD distance learning programs continue to emerge, it is critical to identify and address potential variations in confidence and preparedness of remote learners to enter the pharmacy workforce. 

The rise in popularity of remote or hybrid learning in higher education had been well underway prior to the COVID-19 pandemic. For higher education institutions, remote learning provides several advantages over FTF learning, in particular the accessibility to a more diverse student population to maintain a competitive edge [4,5]. For students, advantages of remote learning include the ability of self-directed and asynchronous learning, content accessibility, and schedule flexibility [6,7,8]. While the benefits of remote learning have been reported, its effectiveness continues to be observed. Remote learning has not been shown to be less effective than FTF learning for undergraduate medical students [9]. Health professions students, including medical, dental, nursing, and health science students, demonstrated positive perceptions and motivation of remote learning [10]. As it relates to pharmacy students, a systematic review consisting of 17 studies revealed online learning improved knowledge and was as effective as traditional learning [11]. In addition, pharmacy students feel comfortable using technology and do not view technology as a barrier for their coursework [12,13].

Student support impacts the students’ learning experience regardless of whether that environment is FTF or remote. Student support can be classified as instructional (assignment instructions, feedback, instructor accessibility, etc.), peer (interaction, etc.), and technical (learning management system navigation, etc.) [14]. The literature shows that student support is closely related to student motivation and learning, while also serving as a foundation for students to achieve course objectives [15,16]. It may be speculated that the type and degree of student support may differ among FTF and remote learners. It has been found that undergraduate students strongly relate interaction, both with instructors and peers, with their perception of support and overall course satisfaction [14]. For educators, it may be useful to assess how FTF support methods translated to remote learning by evaluating students’ perceived confidence and preparedness to enter a career in pharmacy. Cultivating a robust student support structure may result in positive effects on student motivation, learning, and overall course satisfaction. Ultimately, this information could help inform PharmD curricula to better support the educational needs of the remote learner in the future.

At the time of study design, the literature regarding how students perceived the impact of remote learning on their PharmD education and, ultimately, their pharmacy career was limited. Further, the transition to remote educational delivery as a result of the COVID-19 pandemic provided opportunity to survey all students’ perceptions and achieve a larger sample size. Hence, this study was designed to evaluate pharmacy students’ confidence about their communication skills, ability to succeed in a pharmacy career, preparedness for exams, satisfaction with course content delivery, and motivation to participate and study when studying remotely vs. via the FTF mode of instruction at the University of Findlay’s College of Pharmacy. 

## 2. Materials and Methods

The University of Findlay College of Pharmacy follows a 0–6 program structure, where students are directly admitted after high school diploma and complete 2 years of pre-professional study followed by 4 years of professional study before earning a Doctor of Pharmacy (PharmD) degree [17]. Herein, students in the first and second pre-professional years of the pharmacy program are referred to as P1 and P2, respectively, while students in the first through fourth-professional years are referred to as P3 through P6.

Prior to the COVID-19 pandemic, FTF delivery of courses in the pharmacy curriculum was the norm at the University of Findlay. In response to the pandemic, FTF classes moved to an online format for seven weeks during spring 2020 semester and for two weeks during the fall 2020 semester (including one week of final exams during each semester). Starting in fall 2020, if requested by the student, P1 through P5 students enrolled in the College of Pharmacy were offered the ability to participate in pharmacy courses remotely for any duration of the semester. Otherwise, FTF attendance was expected and remote course delivery was reserved for times pursuant to university guidance or case-by-case situations that were excused and approved by the course coordinator.

A survey was emailed to P1 through P6 students (*n* = 292) enrolled at the University of Findlay College of Pharmacy, to evaluate pharmacy students’ confidence about their communication skills, ability to succeed in a pharmacy career, preparedness for exams, satisfaction with course content delivery, and motivation to participate and study when studying remotely vs. via the FTF mode of instruction. The inclusion criteria for receiving the survey was active enrollment in our pharmacy program, and any students not taking pharmacy coursework during spring semester 2021 were excluded from this study. While pharmacy practice skills lab courses were offered remotely part of the time during the year 2020 as well, the survey was designed to analyze students’ overall perceptions regarding the change in the mode of instruction (remote vs. FTF). The April 2021 survey was administered using Google^TM^ Forms. Questions in the survey were adapted from research tools [18,19] and further developed by the investigators. The survey consisted of 10 questions in total; three of the survey’s questions served to collect demographic information, while seven questions measured students’ perceptions of online learning using Likert-scale responses (Appendix A). The latter seven survey questions were generalizable to all courses: didactic, skills-based, and experiential alike. This study was approved for exempt status by the Institutional Review Board (study #1537). Students were provided with an implied consent letter prior to participating in the study; return of the survey was implied consent. No identifiable information was collected from students.

Descriptive statistics were used to analyze demographic data. Investigators used the Kruskal–Wallis test to analyze responses from each of the six pharmacy student cohorts and the Mann–Whitney U test to analyze responses between the P3 (first-professional year) and P6 (fourth-professional year) cohorts specifically with the alpha set at 0.05. Finally, correlation between the responses for survey questions was assessed using the Spearman’s rank correlation coefficient. Power was not calculated; however, 292 students comprised the total pharmacy student population in April 2021, and 10% of the population size (*n* = 30) was considered to be the minimum sample size. SPSS V.25 (IBM Corp., Armonk, NY, USA) was used to perform the Spearman’s rank correlation analysis, Mann–Whitney U test, and Kruskal–Wallis test. Data were marked significant at *p* ≤ 0.05 for all statistical analyses.

## 3. Results

### 3.1. Study Population

**Figure 1:**
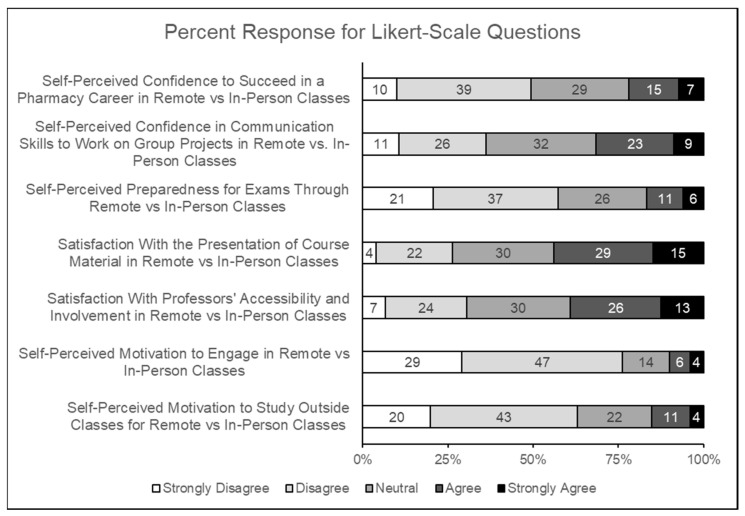
Summary of student responses (%) received for Likert-scale questions from the present survey.

Of the 292 students enrolled at the University of Findlay College of Pharmacy in 2021, 52% completed the survey. Response rates of the various pharmacy student cohorts were as follows: P1 = 49%, P2 = 54%, P3 = 58%, P4 = 54%, P5 = 43%, P6 = 52%. Demographic information is shown in Table 1; the majority of the study’s participants were female and between the ages of 18 and 25 years. Further, the distribution of student participants from each cohort was comparable (Table 1). There was no significant correlation seen between gender and responses for Likert-scale questions regarding confidence, preparedness, motivation, or satisfaction. The majority of the respondents identified their age range to be 18–25 (94.7%), thereby ruling out possible correlation between age and survey responses. Figure 1 summarizes that the responses by study participants for the Likert-scale questions regarding confidence, preparedness, motivation, or satisfaction (comparing remote vs. FTF classes) were either strongly disagree or disagree. The majority of the study participants either strongly disagreed or disagreed with self-perceived confidence, self-perceived preparedness, and self-perceived motivation being higher during remote learning compared with FTF instruction. On the other hand, compared to FTF lectures, the majority of the respondents indicated a greater satisfaction (agreed or strongly agreed) with the presentation of the course material and the professors’ accessibility during remote learning. 

### 3.2. Cohort Trends 

**Figure 2:**
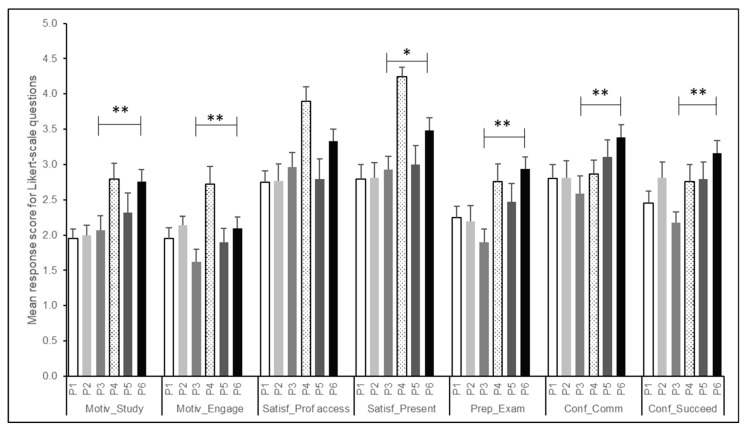
Mean ± SEM responses by student cohorts for Likert-scale questions. Significant differences between the first-professional year (P3) and fourth-professional year (P6) students, as determined by the Mann–Whitney U test, have been indicated for each question on the survey. * *p* ≤ 0.05; ** *p* ≤ 0.01.

Figure 2 summarizes the mean ± SEM responses for the seven Likert-scale questions included in our survey. Interestingly, compared to the pre-professional years and other professional years, students in the first-professional year of our pharmacy program (P3) reported the lowest agreement on 4 out of the 7 Likert-scale survey questions. Students in their second-professional year (P4), surprisingly, reported the highest motivation during and satisfaction with remote learning compared to any other cohort of students. Compared to other cohorts, the P1 students were the most likely to be dissatisfied with the course material through remote learning and were the least motivated to study outside of classes. Compared to the P6 students, the P3 (first-professional year) students had a significantly higher negative opinion (disagreed or strongly disagreed) regarding self-perceived confidence, preparedness, motivation, or satisfaction with remote vs. FTF classes. They were the least likely to be motivated to engage in remote classes, and were more likely to be dissatisfied with the accessibility of professors. Statistically significant differences between responses of the first- and fourth-year professional students, as per the Mann–Whitney U test, are summarized in Figure 2 (* *p* ≤ 0.05); ** *p* ≤ 0.01).

### 3.3. Confidence 

Two questions in the survey assessed student confidence in remote vs. FTF classes. As determined by the Kruskal–Wallis test, Table 2, students’ self-perceived confidence to succeed in a pharmacy career while studying remotely vs. via FTF classes was statistically different among the survey participants from the six cohorts (*p* = 0.01). Students entering their first-professional year of pharmacy school expressed the lowest self-perceived confidence to succeed in a pharmacy career based on learning remotely vs. via FTF instruction. As summarized in Table 3, the Mann–Whitney U test revealed a significant difference in self-perceived confidence to succeed in a pharmacy career between the first-professional and fourth-professional year students (*p* < 0.001), with senior students expressing higher levels of confidence to succeed in the pharmacy career. Furthermore, Spearman’s rank correlation analysis revealed a positive correlation was observed between the cohort level and confidence to succeed in a pharmacy career, indicating higher confidence levels in senior pharmacy students (ρ = 0.210, *p* = 0.01). A positive correlation was also noted between student preparedness for exams and student confidence to succeed in their pharmacy career (ρ = 0.573, *p* < 0.001). 

Students’ self-perceived confidence in their communication skills to work on group projects in remote vs. FTF classes was not statistically different among the six student cohorts (*p* > 0.05; Table 2). Interestingly, P6 students reported a significantly higher level of confidence in their communication skills to work on group projects in remote classes compared to P3 students (Table 3; *p* = 0.008). Importantly, a positive correlation was observed between student confidence about their communications skills and their confidence to succeed in their pharmacy career in remote vs. FTF classes (ρ = 0.254, *p* = 0.002). 

### 3.4. Preparedness 

Students’ self-perceived preparedness for exams in remote vs. FTF classes was significantly different among the six student cohorts (*p* = 0.002). Similar to the trend observed with confidence, the P3 students (first-professional year) expressed the lowest self-perceived preparedness for exams while studying remotely vs. via the FTF mode of instruction. As per the correlation analysis, older students tended to feel more prepared for exams (ρ = 0.270, *p* = 0.001). This correlation was supported by the Mann–Whitney U test, which revealed that P3 students felt significantly less prepared for exams, in remote vs. FTF classes, compared to the P6 student participants (Table 3; *p* < 0.001). Instinctively, a strong positive correlation was noticed between preparedness for exams and other student perception questions, including motivation to study outside of class (ρ = 0.511, *p* < 0.001), motivation to engage in classes (ρ = 0.396, *p* < 0.001), satisfaction with professors’ accessibility and involvement (ρ = 0.445, *p* < 0.001), and satisfaction with course material presentation (ρ = 0.521, *p* < 0.001). Overall, these positive correlations suggest that self-perceived preparedness for exams, in remote classes vs. FTF classes, can be attributed to a high degree of satisfaction and motivation in other surveyed domains of perception.

### 3.5. Satisfaction 

Two questions were included in our survey to determine the level of student satisfaction with professor accessibility and satisfaction with presentation of lecture material in remote vs. FTF classes. As summarized in Table 2, students’ satisfaction with professor accessibility (*p* = 0.001) and satisfaction with presentation of course material (*p* < 0.001) were significantly different among the cohorts for remote vs. FTF classes. Fourth-year professional students expressed a significantly higher level of satisfaction with the presentation of course material in remote vs. FTF classes compared to first-year professional students (Table 3; *p* = 0.05). A positive correlation was observed between student satisfaction with the course material presentation and satisfaction with professors’ accessibility and involvement (ρ = 0.688, *p* < 0.001). Student satisfaction with the course material presentation in remote vs. in-class mode of instruction had a positive correlation with their levels of motivation to engage in remote classes (ρ = 0.296, *p* < 0.001), their motivation to study outside the class (ρ = 0.338, *p* < 0.001), and their perceived confidence to succeed in pharmacy career (ρ = 0.416, *p* < 0.001). 

### 3.6. Motivation 

As part of this survey, 2 Likert-scale questions were included to assess self-perceived motivation among pharmacy students. Results from the Kruskal–Wallis test, summarized in Table 2, suggests that the motivation to engage in lectures (*p* = 0.002) and motivation to study outside of classes (*p* = 0.003) in remote vs. FTF classes were significantly different among the six cohorts. A significantly higher motivation to study outside class in remote classes (Table 3; *p* = 0.008) and motivation to engage in remote classes (Table 3; *p* = 0.008) was reported by fourth-professional year students compared to first-professional year students. Furthermore, a positive correlation was observed between cohort and motivation to study outside of class, with senior students likely to report higher motivation levels (ρ = 0.281, *p* < 0.001), which supports the notion that progression in the program and increased college experience may positively impact motivation. Students who were satisfied with the accessibility of their professors (ρ = 0.268, *p* = 0.001) and satisfied with the presentation of the course material (ρ = 0.338, *p* < 0.001) were more likely (positive correlation) to report higher motivation levels to study outside the class. Instinctively, students reporting higher motivation to engage in online classes also reported higher motivation levels to study outside the class as well (ρ = 0.501, *p* < 0.001). 

## 4. Discussion

The results from the present study showed that pharmacy students’ confidence about their ability to succeed in a pharmacy career, preparedness for exams, satisfaction with course content delivery and professors’ accessibility, and motivation to participate and study between remote and FTF mode of instruction were significantly different among the six cohorts. More specifically, students in the first professional year of the program reported significantly lower levels of confidence in their communication skills, confidence in their ability to succeed in a pharmacy career, preparedness for exams, satisfaction with the presentation of course material, and motivation to study and engage in remote vs. FTF classes compared to students in the fourth professional year of the program. Interestingly, P3 students’ satisfaction with professors’ accessibility and involvement in remote vs. FTF classes was comparable to that of P6 students. Second-professional year students, surprisingly, had the most positive responses regarding motivation during and satisfaction with remote education compared to other cohorts. Furthermore, the majority of responses by study participants for the Likert-scale questions regarding confidence, preparedness, or motivation (comparing remote vs. FTF classes) were either strongly disagree or disagree. Students, however, reported higher level of satisfaction both with the presentation of the course material as well as the professors’ accessibility in remote classes vs. FTF instruction. In particular, students’ motivation was the most significantly impacted, as demonstrated by the highest number of strongly disagree and disagree responses for both engaging in classes and studying outside of classes. In the wake of the COVID-19 pandemic, results of students’ perceptions regarding the transition to remote classes provides opportunities for educators.

The results of this study further show that students’ self-perceived preparedness was strongly correlated with self-perceived confidence, motivation, and satisfaction. For example, students who were satisfied with how the course material was presented were more likely to be satisfied with the instructor’s involvement in the course and feel prepared for exams. Additionally, students who felt prepared for exams exhibited confidence to succeed in their future pharmacy career. Based on these results, focusing on further developing instructional support, such as providing additional touchpoints between instructors and students [14], may help improve students’ perceptions in remote learning. While it can be anticipated that the methods will vary depending on the course, such as skills labs compared to traditional didactic courses, providing additional instructional support may positively impact students’ satisfaction, preparedness, and career confidence regardless. 

Instructors may further promote a positive online learning experience by focusing on ways to increase students’ motivation inside and outside of the virtual classroom. Lack of motivation, particularly following the onset of the pandemic, has been reported among pharmacy [20] and college students in general [21]. Our results suggest that students who felt motivated to engage in class also felt motivated to study outside of class. Further, students who were motivated to study outside of class felt prepared for exams. In light of these results, educators can benefit from considering strategies to promote student engagement in online classes. Fostering peer and instructional support could be one way to accomplish this, such as by arranging peer study groups, assigning group projects, and facilitating discussion boards [13,14,22]. Providing opportunities to enhance engagement in online courses may empower students to study outside of class and ultimately feel prepared for upcoming exams.

We observed significant differences in responses to the majority of Likert-scale questions among all cohorts and specifically between the P3 and P6 students. The cohort year itself may be just one piece of the puzzle, considering students have varying experiences and circumstances that may have influenced their responses. This may be explained by students acquiring an enhanced understanding of the expectations and the study time that is necessary for exams as experience in college courses increases. This finding supports previous studies that have shown that the less college experience students have, the more difficult the transition is to confidently adjust to remote learning [23]. Similarly, a reduction in medical students’ perception of career preparedness was also seen during the switch to remote learning [24]. Based on our observations, though, students’ negative perceptions improved as they progressed through the curriculum, demonstrated by overall more positive responses from P4, P5, or P6 students compared to those of P3 students. This observation is supported by findings from another study which noted that, compared to other years, the first-professional year students are least prepared to tackle remote learning [25]. While not examined in the present survey, the work status and the amount of work per week for P1 students during the pandemic may have been attributed to elevated stress in these students [26], thereby attributing to the observed lack of confidence and preparedness in our survey results. Taking this into consideration, pharmacy educators may designate more time and instructional support to P3 students in an effort to improve students’ perceptions of confidence and preparedness.

As previously mentioned, our study observed that P3 self-perceptions are disproportionately reduced compared to those of the other cohorts. The mental struggles of university students have been documented; pharmacy students have been reported to experience depression and anxiety [27]. Further, the mental struggles of students in the first-professional year of pharmacy program, owing to the pandemic, have been documented previously [28]. Limited college course experience and the introduction of practical-based competencies may be responsible for this negative impact on student self-perceptions [13]. In fact, a recent study has documented a comparable level of stress in students, prior to and during the pandemic, before starting their performance-based assessments in skills labs [29]. Practical-based examinations may require additional support in conjunction with the online curriculum for students to feel adequately prepared for activities of that nature. This is supported by a prior study completed during the COVID-19 switch to remote learning, which found that students did not feel prepared for practicals solely based on the online curriculum, despite feeling that the courses were proficient at teaching material without losing content [30]. A recent study has highlighted the fact that first-professional year students may observe a decline in their habits to spend time on assigned reading and study the course material daily [31] which, in the context of the pandemic, may have exacerbated their negative perceptions as assessed in the present survey. Based on these observations, first-professional year students may benefit from additional course structure and communication in remote classes in light of their lacking previous college course experience, engagement in classes, and preparation for coursework.

Our study had a few limitations. Considering the survey was available for completion during April 2021, the responses reflect a snapshot of students’ perceptions of remote vs. FTF learning. There is also the potential of recall bias, as students completed the survey months after remote learning was mandatory, as well as selection bias, since our study relied on students’ voluntary participation. Our survey was also limited in the sense that it only reflects 9 weeks of online learning in total, in accordance with university-mandated procedure, and did not assess factors that may have influenced students’ perceptions of their learning experience, such as stress from family, work, or pandemic-related situations. Future studies could be conducted to evaluate students’ perceptions of remote learning in a post-pandemic world and compared to this study’s findings. In addition, students’ experience with college course delivery and modality may have influenced their perceptions. At the time our survey was administered, P1 students had little-to-no experience of college courses in a FTF format, compared to P6 students with years of FTF college course experience to compare to remote learning. Similarly, the timing of the pandemic may have influenced students’ perceptions, considering P1 and P2 students faced the combined uncertainty of starting college courses during a pandemic, in contrast to students who had completed years of the program by the time the pandemic started. The inability to measure the potential impact of these factors was a weakness of our survey design. Strengths of our study on the other hand include the robust response rate from the pharmacy student population, fair representation of each cohort, and insight into how students perceive online education in a professional pharmacy program. Future research may involve several schools or colleges of pharmacy to assess trends or evaluate for differences in students’ responses, particularly programs with newly introduced online or hybrid programs in addition to traditional FTF methods, to better understand where instructors can make targeted interventions to support online learning.

## 5. Conclusions

In conclusion, the present study demonstrates that while students’ perceptions regarding remote learning improved as they progressed through the curriculum, there is an opportunity for educators to better support students during their first-professional year. Additionally, our results indicate that didactic approaches which promote student motivation during remote classes can improve student motivation to study outside class and to prepare for assessments. 

## Figures and Tables

**Table 1:** Demographic details of study participants.

Variable	*n*	%
**Gender**		
Male	42	27.8
Female	109	72.2
**Age**		
18–25	143	94.7
26–30	5	3.3
31–35	2	1.3
>35	1	0.7
**Pharmacy Cohort**		
P1	20	13.2
P2	21	13.9
P3	29	19.2
P4	29	19.2
P5	19	12.6
P6	33	21.9

**Table 2:** Survey responses to Likert-scale questions by student cohorts (P1–P6). The level of significance for Kruskal–Wallis Test has been included for each question.

Self-Perceived Confidence to Succeed in a Pharmacy Career in Remote vs. FTF Classes							*p*-Value (Kruskal–Wallis Test)
Likert-Scale Response	Pharmacy Cohort						
	P1	P2	P3	P4	P5	P6	**0.01**
(1) Strongly Disagree	1	1	5	6	1	1	
(2) Disagree	11	8	16	7	8	9	
(3) Neutral	6	8	7	7	6	9	
(4) Agree	2	2	0	6	2	10	
(5) Strongly Agree	0	2	1	3	2	3	
**Self-Perceived Confidence in Communication Skills to Work on Group Projects in Remote vs. FTF Classes**							
(1) Strongly Disagree	0	3	7	3	2	1	0.08
(2) Disagree	10	5	9	7	3	4	
(3) Neutral	4	7	7	12	5	13	
(4) Agree	6	5	1	5	9	8	
(5) Strongly Agree	0	1	5	2	0	5	
**Self-Perceived Preparedness for Exams Through Remote vs. FTF Classes**							
(1) Strongly Disagree	2	5	12	6	4	2	**0.002**
(2) Disagree	12	10	11	8	6	8	
(3) Neutral	5	4	4	6	6	14	
(4) Agree	1	1	1	5	2	6	
(5) Strongly Agree	0	1	1	4	1	2	
**Satisfaction With the Presentation of Course Material in Remote vs. FTF Classes**							
(1) Strongly Disagree	0	1	2	0	3	0	**<0.001**
(2) Disagree	10	8	6	1	2	6	
(3) Neutral	3	7	13	2	7	12	
(4) Agree	6	4	4	15	6	8	
(5) Strongly Agree	0	1	2	11	1	7	
**Satisfaction With Professors’ Accessibility and Involvement in Remote vs. FTF Classes**							
(1) Strongly Disagree	1	3	1	0	4	1	**0.001**
(2) Disagree	5	6	11	5	4	5	
(3) Neutral	12	6	8	4	4	12	
(4) Agree	2	5	6	9	6	12	
(5) Strongly Agree	0	1	3	11	1	3	
**Self-Perceived Motivation to Engage in Remote vs. FTF Classes**							
(1) Strongly Disagree	5	2	18	5	7	7	**0.002**
(2) Disagree	11	14	7	11	8	20	
(3) Neutral	4	5	1	4	3	4	
(4) Agree	0	0	3	5	1	0	
(5) Strongly Agree	0	0	0	4	0	2	
**Self-Perceived Motivation to Study Outside Classes for Remote vs. FTF Classes**							
(1) Strongly Disagree	4	4	11	4	5	2	**0.003**
(2) Disagree	13	13	10	9	8	12	
(3) Neutral	3	4	3	8	2	13	
(4) Agree	0	0	5	5	3	4	
(5) Strongly Agree	0	0	0	3	1	2	

**Table 3:** Results of the Mann–Whitney U Test comparing student responses to Likert-scale questions in the first-professional year (P3) and fourth-professional year (P6) of PharmD curriculum.

Questions	Cohort	*n*	Mean Rank	Sum of Ranks	Z	Mann–Whitney U Test (*p* Value)
Self-Perceived Confidence to Succeed in a Pharmacy Career in Remote vs. FTF Classes	P3	29	22.67	657.5	−3.661	**<0.001**
	P6	32	38.55	1233.5		
Self-Perceived Confidence in Communication Skills to Work on Group Projects in Remote vs. FTF Classes	P3	29	24.5	710.5	−2.651	**0.008**
	P6	31	36.11	1119.5		
Self-Perceived Preparedness for Exams Through Remote vs. FTF Classes	P3	29	22.03	639	−3.892	**<0.001**
	P6	32	39.13	1252		
Satisfaction With the Presentation of Course Material in Remote vs. FTF Classes	P3	27	25.89	699	−1.941	**0.05**
	P6	33	34.27	1131		
Satisfaction With Professors’ Accessibility and Involvement in Remote vs. FTF Classes	P3	29	27.88	808.5	−1.541	0.12
	P6	33	34.68	1144.5		
Self-Perceived Motivation to Engage in Remote vs. FTF Classes	P3	29	25.48	739	−2.668	**0.008**
	P6	33	36.79	1214		
Self-Perceived Motivation to Study Outside Classes for Remote vs. FTF Classes	P3	29	25.26	732.5	−2.654	**0.008**
	P6	33	36.98	1220.5		

## Data Availability

Data are available in Figure 1 and Figure 2 and Table 1, Table 2 and Table 3. Individual reported results can be made available by request to the corresponding author.

## Appendix A

**Please select your gender.**				
Female		Male		Other
**Please select your pharmacy cohort:**				
P1 (Year 1)		P2 (Year 2)		P3 (Year 3)
P4 (Year 4)		P5 (Year 5)		P6 (Year 6)
**Please select the age range that best describes your age:**				
Less than 18	18–25	26–30	31–35	Greater than 35
**Please rate your level of agreement with the following statements from strongly disagree (1) to strongly agree (5).**				
I feel more motivated to study outside of class for remote classes vs. FTF classes				
1	2	3	4	5
I feel more motivated to engage in remote classes vs. FTF classes				
1	2	3	4	5
I feel as satisfied with my professors’ accessibility and involvement in remote classes vs. FTF classes				
1	2	3	4	5
I feel as satisfied with the course material presentation in remote classes vs. FTF classes				
1	2	3	4	5
I feel sufficiently prepared for exams through remote classes vs. FTF classes				
1	2	3	4	5
I am as confident in my communication skills to work on group projects for remote classes vs. FTF classes				
1	2	3	4	5
I am as confident I can succeed in pharmacy based on my learning from remote classes vs. FTF classes				
1	2	3	4	5
